# Health anxiety among Tunisian nursing students: A Cross-Sectional Study

**DOI:** 10.1192/j.eurpsy.2026.11224

**Published:** 2026-07-31

**Authors:** R. Masmoudi, D. Triki, F. Guermazi, N. Regaieg, I. Feki, J. Masmoudi, I. Baati

**Affiliations:** Psychiatry A, Hedi Chaker university hospital, Sfax, Tunisia

## Abstract

**Introduction:**

Nursing students are intensively exposed to medical knowledge and clinical environments, which may predispose them to health anxiety (HA), a condition characterized by excessive fear of having a serious illness.

**Objectives:**

To assess the prevalence of HA symptoms and to determine associated sociodemographic and clinical factors among nursing students in Tunisia.

**Methods:**

A descriptive and analytical cross-sectional study was conducted in February 2025. An exhaustive sampling method was used, soliciting all 340 undergraduate nursing students across three academic years at the institute. Data were collected via an online questionnaire.

The Health Anxiety Questionnaire (HAQ) was used to understand behaviors such as reassurance seeking, health worry and preoccupation, fear of illness and death, and obstruction with life.

**Results:**

The final sample included 184 students (complete response rate: 49.6%). The mean age was 20.4±1.1 years, with a balanced gender distribution (50.5% female). The prevalence of significant HA symptoms (HAQ≥20) was 71.2%. Alarmingly, 44% of the total sample had scores indicating probable clinical hypochondria (HAQ≥30). Analysis of the HAQ subscales revealed high scores for **Health Worry (10.57 ± 4.74)** and **Fear of Illness/Death (9.29 ± 4.28)**.

Bivariate analyses identified two factors significantly associated with higher HAQ scores: **age < 20 years (p=0.003)** and a **personal history of psychiatric disorders (p=0.023)**.

No significant association was found with gender, academic year, or family history of medical conditions.

**Conclusions:**

This study reveals a high prevalence of health anxiety among Tunisian nursing students. A targeted intervention to create support systems is needed within educational frameworks to mitigate this significant burden of health anxiety, focusing particularly on identified at-risk groups.

**Disclosure of Interest:**

None Declared

